# Formalin-Fixed Paraffin-Embedded (FFPE) samples are not a beneficial replacement for frozen tissues in fetal membrane microbiota research

**DOI:** 10.1371/journal.pone.0265441

**Published:** 2022-03-17

**Authors:** Rochelle Hockney, Caroline H. Orr, Gareth J. Waring, Inge Christiaens, Gillian Taylor, Stephen P. Cummings, Stephen C. Robson, Andrew Nelson

**Affiliations:** 1 School of Health, Leeds Beckett University, Leeds, United Kingdom; 2 School of Health and Life Sciences, Teesside University, Middlesbrough, United Kingdom; 3 National Horizons Centre, Teesside University, John Dixon Lane, Darlington, United Kingdom; 4 Institute of Cellular Medicine, Newcastle University, Newcastle, United Kingdom; 5 Faculty of Health and Life Sciences, Northumbria University, Newcastle, United Kingdom; BC Children’s Hospital, CANADA

## Abstract

Formalin-Fixed Paraffin-Embedded (FFPE) tissues are routinely collected, archived, and used for clinical diagnosis, including maternal and neonatal health. Applying FFPE samples to microbiota research would be beneficial to reduce preparation, storage and costs associated with limited available frozen samples. This research aims to understand if FFPE fetal membrane samples are comparable to frozen tissues, which are the current gold standard for DNA microbiota analysis. Extracted DNA from nine matched paired patients were sequenced by Illumina sequencing of the V4 16S rRNA gene region. This included duplicate frozen amnion and chorion fetal membrane rolls or FFPE combined amniochorionic samples. Negative controls of surrounding wax blocks and DNA extraction reagents were processed alongside samples using identical methods. DNA quality and quantity was assessed by NanoDrop, agarose gel electrophoresis and Bioanalyzer. Decontam and SourceTracker were integrated into microbiota analysis to identify the presence of contaminating sources. The bacterial profile and nine genera differed between FFPE and frozen fetal membranes. There were no differences in bacterial profiles between FFPE samples and corresponding wax negative controls, with 49% of bacteria in FFPE fetal membrane samples matched to the source origin of paraffin wax, and 40% originating from DNA extraction reagent sources. FFPE samples displayed high fragmentation and low quantity of extracted DNA compared to frozen samples. The microbiota of FFPE fetal membrane samples is influenced by processing methods, with the inability to differentiate between the microbiota of the tissue sample and the surrounding wax block. Illumina sequencing results of FFPE and frozen fetal membrane samples should not be compared using the methods employed here. Variation could be influenced by limitations including storage time, DNA extraction and purification methods. To utilise FFPE fetal membrane samples in microbiota research then contamination prevention and detection methods must be included into optimised and standardised protocols, with recommendations presented here.

## Introduction

Placental morphology is routinely investigated in a clinical setting using Formalin-Fixed Paraffin-Embedded (FFPE) placental and fetal membrane tissues, this including for the diagnosis of histological chorioamnionitis (HCA) [1]. Following histological analysis and diagnosis, these FFPE samples are stored but infrequently reused. Frozen tissues can sometimes be collected simultaneously, for example, as routine procedure for the Newcastle Uteroplacental Tissue Biobank [2].

Frozen tissues are utilised as the current gold standard tissue preparation method for microbiological studies, due to high quality, low damaged DNA [3–5]. The use of FFPE samples in microbiological research would be beneficial due to the routine collection and availability of samples, plus ease of retrospective and repeat analysis, especially when frozen samples are not available due to limited sample volumes or storage capacity [3,4,6]. Utilising one tissue type for clinical diagnosis and microbiota research would be preferable to avoid duplication, plus reduce processing requirements and storage costs [7]. This would give the ability to accurately link molecular findings to pathological and clinical diagnosis. Although initiatives such as project PLACENTA aims to encourage the biobanking of reproductive tissues, samples remain underrepresented due to low funding and increased maintenance [7]. Immediate processing and snap freezing of reproductive samples is not always possible in the clinical or home birth setting [8]. Thus, it would be beneficial to utilise the readily available, remnant FFPE samples for clinical research.

FFPE samples have been successfully utilised in sequencing and qPCR research, yet the success of this varies by processing and storage duration. The harsh fixative process creates a tough physical barrier, as the formalin reacts with amino groups and adds a methylol group, which increases cross-linkage between amino acids leading to DNA degradation and fragmentation [9,10]. This may negatively impact downstream methodology unless correct protocols are employed. Archived ovarian FFPE samples display an 90% success rate in whole exome sequencing, however, extract fragment size reduced with increasing storage time of 32 years [11]. Conversely, archived FFPE samples stored for >100 years produced minimal difference in DNA damage throughout storage duration when analysed by Illumina HiSeq [12]. Three to seven years has recently been suggested as an amended maximum storage duration to ensure optimum results [5,13].

The comparative analysis of FFPE and frozen tissues has been performed previously from healthy or cancerous tissues, including colon, breast and brain biopsies [9,14–16]. But investigation into the fetal membrane microbiota using 16S rRNA gene sequencing technology and DNA quality analysis specifically comparing FFPE and frozen tissue samples is limited. Similar gene expression profiles from FFPE and frozen breast tissue have been observed, with the ability to correctly identify changes in tumour grades from FFPE samples with comparable accuracy to frozen tissues [14]. Additional research has suggested that using a selective bacterial marker gene ensures improved amplification of DNA extracted from FFPE colorectal samples [16].

Low abundance and low biomass samples, such as fetal membranes have been criticised for reduced accuracy in microbiota research [3,17,18], as sample source signals are more susceptible to being concealed by contaminating sequences, thus masking the tissue sample microbiota [12,19]. This combined with the complex processing of FFPE samples, means that detecting external contaminating bacteria is important. For this reason, the RIDE minimum standards checklist for low microbial biomass studies aims to reduce experimental bias and investigate contamination [20]. The checklist includes comparing negative controls to biological samples including sample blanks, DNA extraction blanks and no template controls within each batch; exploring and interpreting the impact of contaminating taxa on results within the study; and reporting measures used to reduce and assess contamination [20]. Alongside this checklist, multiple post-processing computational sequencing analysis methods aim to overcome the limitation of microbial contamination within low biomass material, including Decontam, SourceTracker and combined filtering methods [19,21–23]. Employing these specialised workflows has highlighted the potential and beneficial use of FFPE tissues in cancerous, vaginal, oral, and placental tissues [21,24–27].

### Aim

The aim of this research was to determine if routinely collected and archived FFPE fetal membrane samples are comparable to frozen amniochorionic tissues as the current sample of choice for fetal membrane microbiota analysis.

## Methods

### Sample selection and processing

FFPE fetal membrane rolls consisting of a combined coil of amniochorionic membranes, plus corresponding individual frozen amnion and chorion were collected from nine patients, with informed consent.

Sample collection, diagnosis and storage of the frozen subset are described in Waring *et al* (2015) [28]; the original prospective study to investigate inflammatory signalling in the fetal membranes, and Hockney *et al* (2020); an additional retrospective study investigating the microbiota of the fetal membranes with a negative maternal inflammatory condition [29]. FFPE samples were prepared as per routine clinical protocols. Combined unseparated amniochorionic membrane rolls (4–5 cm) were fixed in 10% neutral buffered formalin for 24 hours, embedded in paraffin wax, sectioned to 3 μm slices, stained with haematoxylin and eosin (H&E) and histologically examined by an independent clinician. FFPE amniochorionic rolls were stored within a sealed cassette case at room temperature for three years before use in this retrospective study. Further sampling methods were followed as described previously [28,29]. All patient samples were from spontaneous preterm birth with inflammatory HCA (n = 9), as our previous research indicated that these samples were more likely to contain a microbiota representative of the tissue sample detectable over contamination levels [29]. Samples were processed in triplicate, with individual amnion and chorion results from frozen analysis merged to closer represent the FFPE combined amniochorionic fetal membrane rolls. Nine patients with duplicate frozen or FFPE stored tissues, processed in triplicate provided 54 samples for analysis. Triplicate extractions and products were merged during visualisation of results.

### Genomic DNA extraction

Prior to DNA extraction, FFPE samples were sectioned into 3 x 10 μm sections using a manual rotary microtome (Leica, RM2125). External slices were discarded to minimise the impact of any environmental contamination transferred during storage. The microtome and blades were cleaned between patient samples to avoid cross contamination.

Genomic DNA was extracted from 25 mg frozen samples using QIAamp Fast DNA Tissue Kit (Qiagen), and 25 mg FFPE samples were processed using BiOstic FFPE tissue DNA isolation kit (MoBio), both as per manufacturers protocols. Both kits utilised bead-based methods for mechanical lysis, alongside enzymatic and chemical lysis provided by the kit reagents. The DNA extraction kits also used silica membranes for DNA purification before washing and eluting. Further DNA extraction protocols can be found in supporting information (S1 Protocols). Eluted nucleic acids were stored at -20°C until required for analysis (maximum duration: three months).

Due to processing low biomass materials, multiple negative controls were used to adhere to the RIDE guidelines [20]. Due to the use of different kits, respective negative controls were taken per batch from the Qiagen frozen kit (n = 9) or MoBio FFPE kit (n = 18). Different kits were used due to the independent tissue characteristics and sample preparation methods, including harsh enzymatic buffers and chemical required for deparaffinisation of FFPE samples. For the FFPE subset, additional controls were taken from the surrounding embedded paraffin wax adjacent to the tissue to identify the microbiota of infiltrating wax. All remaining methodology and reagents remained consistent. All sample preparation stages were performed in low contamination control environments and under sterile techniques. Library preparation, pre-PCR and post-PCR steps were performed in a clean room and within separate UV cabinets. Samples were processed in a random order to avoid batch effects.

Prior to microbial analysis, the quantity and quality of eluted DNA were assessed by NanoDrop 1000 Spectrophotometer (V3.8.1 Thermo Scientific) and agarose gel electrophoresis. Bioanalyzer chip-based electrophoresis (Agilent 2100) was used to further investigate the quality and quantity of FFPE extracted DNA. Detailed quantitative and qualitative methods can be found in the supporting information (S1 Protocols).

### Illumina amplicon sequencing

Amplicon sequencing of the V4 region of the 16S rRNA gene was performed by NU-OMICs (Northumbria University, UK), as described previously [30–32]. The short 250 bp V4 region was targeted to achieve optimum results from fragmented FFPE DNA, plus allow comparison to previous publications [28,29]. Briefly, 1 μl of samples, wax controls or kit negatives were amplified using the 16S rRNA gene primer specific to the V4 region. Products were normalised, pooled and purified via AMPure XP beads. Library quality was assessed using a Bioanalyzer and quantified by qPCR. Paired end bridge amplification with sequencing by synthesis was performed on samples using the Illumina MiSeq and MiSeq reagent kits. Each plate also contained a blank (dH_2_O), negative no template control and positive control of microbial DNA standard (2000ng, diluted 1 in 10, ZymoBIOMICS). The mock microbial standard functions as an in-run quality control, containing highly diverse bacterial coverage [20]. Further Illumina amplicon sequencing protocols can be found in the supporting information (S1 Protocols).

### Microbiota analysis

Following amplicon sequencing FastQ files were processed through DADA2 1.4 [31] and Bioconductor (Version 2) [30] in R [33]. FastQ files were trimmed and filtered to remove low quality reads (<30 Q score) and clustered into Amplicon Sequence Variants (ASVs). Chimeras were removed by *de novo* removal and low read count features with <2 reads were removed before assigning taxonomy using RDP14 reference database [34].

Filtered samples were processed through Phyloseq [35] and Microbiome Analyst [36,37] for abundance (relative and absolute), diversity (GUniFrac, PCoA by Bray-Curtis PERMANOVA) and univariate analysis (Mann-Whitney or Kruskal-Wallis), with p-value false discovery rate corrections (FDR). FDR aims to control for errors arising due to multiple simultaneous comparisons. Results were further analysed by Kruskal-Wallis and Pairwise Wilcoxon Rank Sum in R and visualised with ggplot2 [33].

### Decontam and SourceTracker

Further data analysis included reporting and comparing taxa detected in samples, wax block controls and kit regent negative controls. Decontam in R [21,33] was firstly utilised to detect and report contamination from sequencing data but avoid the removal of ASVs originating in samples [21]. This method is preferable when samples are of low abundance with the possibility that contamination may dominate the microbiota [21]. SourceTracker (1.0.1) [23,33] was used to determine the proportion of contribution to the sample microbiota from sources of negative controls, including sources of DNA extraction kit reagents and respective wax controls. SourceTracker compares each ‘source’ site to ‘sink’ sites to determine the community composition [23]. Results from Decontam and SourceTracker were tabulated, plus per patient pie charts created for presentation of SourceTracker results.

## Results

### Matched paired FFPE and frozen fetal membrane bacterial community composition

**Fig 1:**
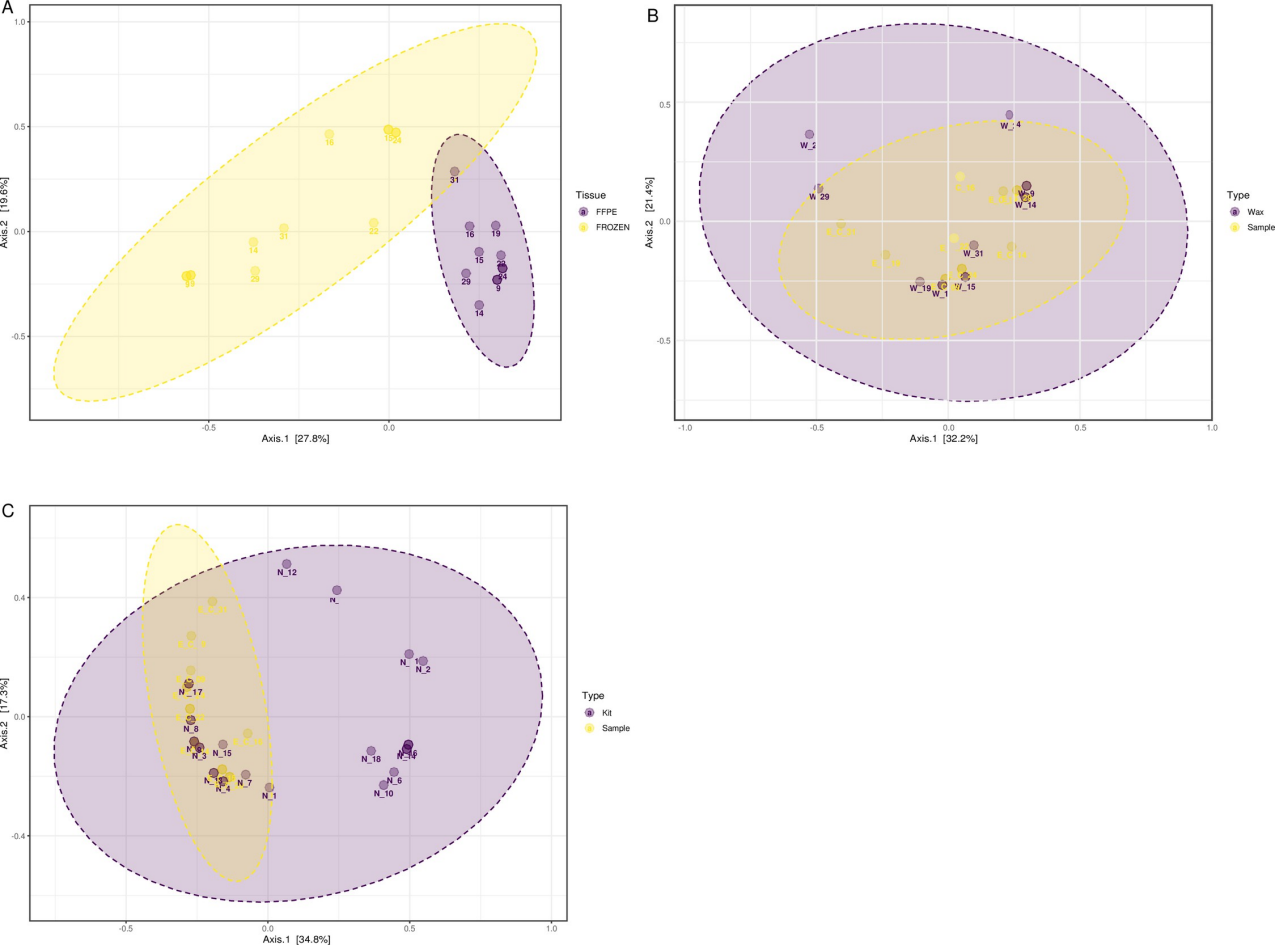
PCoA analysis of frozen and FFPE fetal membrane samples and processed negative controls. PCoA plots analysed from V4 16S rRNA amplicon sequencing of DNA extracted from Formalin-Fixed Paraffin-Embedded (FFPE) fetal membrane samples and frozen stored fetal membrane tissues (A). FFPE samples compared to corresponding surrounding paraffin wax negative controls (B), and additionally FFPE samples compared to DNA extraction kit reagent negative controls (C).

Duplicate samples were taken from matched paired FFPE and frozen fetal membrane tissue samples. Patient demographics (n = 9) are listed in in the supporting information (S1 Table). Distinct clustering of FFPE or frozen tissues were detected from PCoA, with samples clustering by tissue preparation and storage method, whereas duplicate patient samples did not cluster together (R^2^ = 0.149, p = 0.012; Fig 1A). Throughout analysis FFPE tissue samples were also compared to negative controls of surrounding paraffin wax and DNA extraction kit negative regents. PCoA displayed an overlap and no distinction between FFPE samples and wax negative controls (R^2^ = 0.085, p = 0.184; Fig 1B). No distinct clusters were also detected when comparing FFPE samples to extraction kit negative controls (R^2^ = 0.007, p = 0.163; Fig 1C).

**Fig 2:**
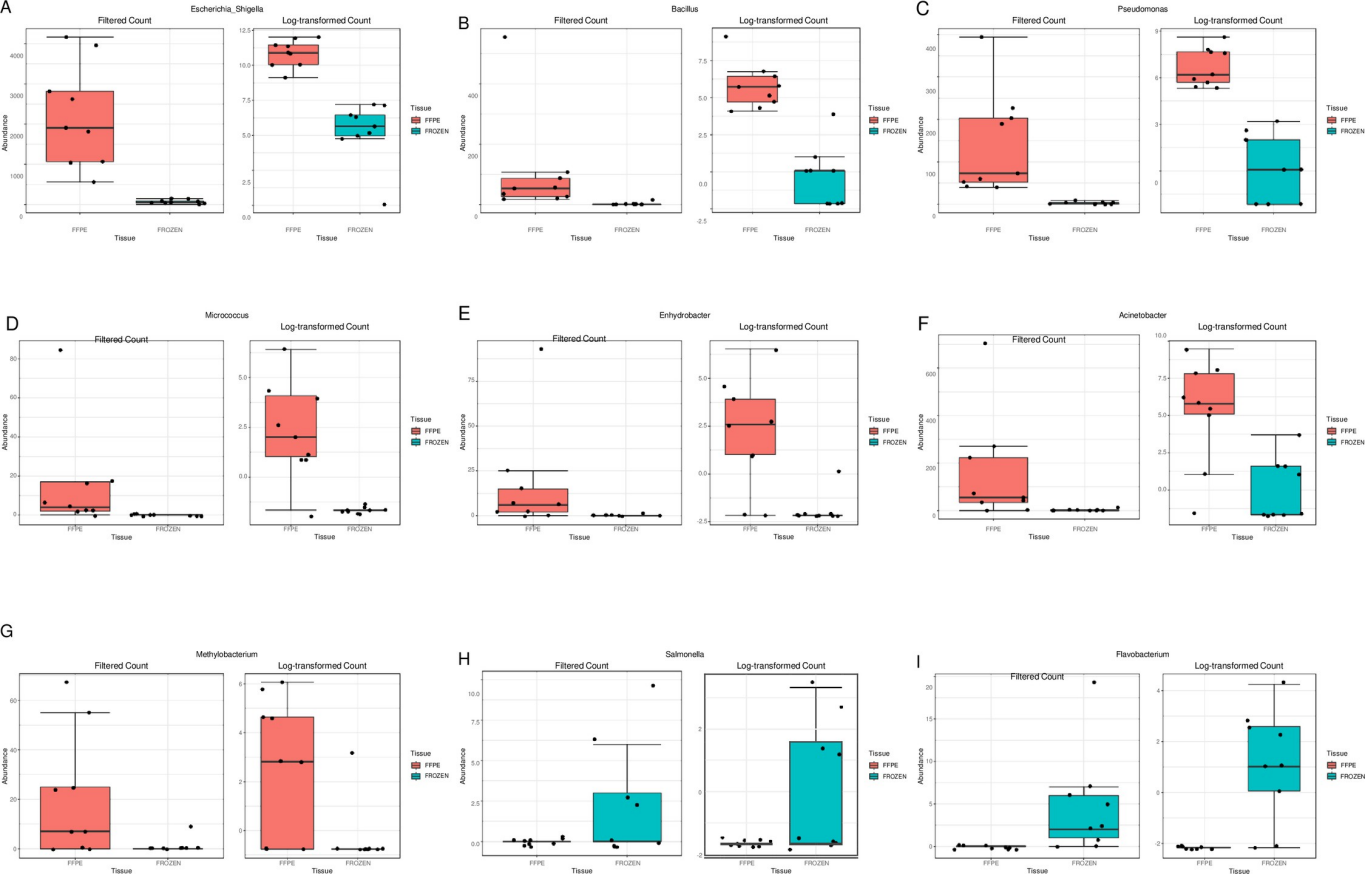
Genera with significantly different read counts between FFPE and frozen fetal membrane samples. Plots created for nine genera which were detected as significantly different via univariate analysis between Formalin-Fixed Paraffin-Embedded (FFPE) fetal membrane samples and frozen stored fetal membrane tissues. Data presented by read count and log transformed.

Univariate analysis at the genera level highlighted nine genera significantly different between FFPE and frozen fetal membrane samples. Those with significantly greater read counts from FFPE samples were *Escherichia/Shigella*, *Bacillus*, *Pseudomonas*, *Micrococcus*, *Enhydrobacter*, *Acinetobacter* and *Methylobacterium* (Table 1). Individual read count plots can be seen in Fig 2. *Micrococcus* was not detected on frozen stored samples, with a read count of 136 from FFPE samples. However, most *Micrococcus* contribution detected in FFPE samples were from one patient (FFPE 09 = 85/136 reads; Fig 2D). Significantly greater read counts for *Salmonella* (12 vs 0, p = 0.038; Fig 2H) and *Flavobacterium* (42 vs 0, p = 0.021; Fig 2I) were detected on frozen fetal membranes and not found on FFPE samples. Further patient specific variance was detected. FFPE sample 09 had the greatest read count for *Micrococcus*, *Enhydrobacter*, *Acinetobacter* and *Methylobacterium*, whereas two FFPE samples had no read count detected for this genus. Whereas FFPE sample 31 had the greatest read counts for *Escherichia/Shigella*, *Bacillus* and *Pseudomonas*. Similar results were also presented by the two genera which were significantly greater in frozen samples, with *Salmonella* having the greatest read count from frozen sample 24 and *Flavobacterium* from frozen sample 19. These two genera were also not detected in three frozen patient samples, however *Flavobacterium* and *Salmonella* were never detected from FFPE fetal membrane samples. *Escherichia/Shigella* was detected as the most prominent genus within FFPE samples (36%), compared to 8% detected in frozen samples (S1 Fig) *Ureaplasma* (28%) and *Prevotella* (26%) had the greatest abundance in frozen samples, compared to 1% and 10% in FFPE samples (S1 Fig).

**Table 1:** Read count comparison between sample type and contamination controls.

Genus	FFPE VS Frozen tissues	FFPE VS Wax controls	FFPE VS Kit negative controls
*Escherichia/Shigella*	0.002*	0.938	0.026*
*Bacillus*	0.007*	0.849	0.021*
*Pseudomonas*	0.007*	0.849	0.021*
*Micrococcus*	0.019*	0.430	0.002*
*Flavobacterium*	0.021*	0.430	0.026*
*Enhydrobacter*	0.038*	0.063	0.014*
*Salmonella*	0.038*	0.934	0.445
*Acinetobacter*	0.038*	1.000	0.044*
*Methylobacterium*	0.044*	0.687	0.055
*Lactobacillus*	0.300	0.430	<0.001**
*Bacteroides*	0.074	0.456	0.002*
*Blautia*	0.106	0.430	0.002*
*Ruminococcus*	0.415	0.456	0.002*
*Coprococcus*	0.109	0.430	0.002*
*Dietzia*	0.109	0.063	0.006*
*Staphylococcus*	0.098	0.430	0.011*
*Collinsella*	0.563	0.670	0.013*
*Dorea*	0.450	0.430	0.013*
*Gemmiger*	0.415	0.430	0.017*
*Prevotella*	0.726	0.430	0.019*
*Streptococcus*	0.152	0.690	0.019*
*Alistipes*	0.152	0.430	0.021*
*Corynebacterium*	0.114	0.700	0.021*
*Not_Assigned*	0.070	0.586	0.021*
*Anaerostipes*	0.625	0.719	0.025*
*Faecalibacterium*	0.387	0.552	0.026*
*Clostridium_XI*	0.405	0.700	0.027*
*Roseburia*	0.109	0.634	0.030*

Significant differences in read counts presented at the genera level, compared between matched paired Formalin-Fixed Paraffin-Embedded (FFPE) and frozen fetal membrane sample preparations, or FFPE samples and wax negative controls, or FFPE samples and DNA extraction kit negative controls. Significance determined via Kruskal-Wallis with false discovery rate (FDR) corrections. P-value displayed to <0.05* or <0.001**.

**Fig 3:**
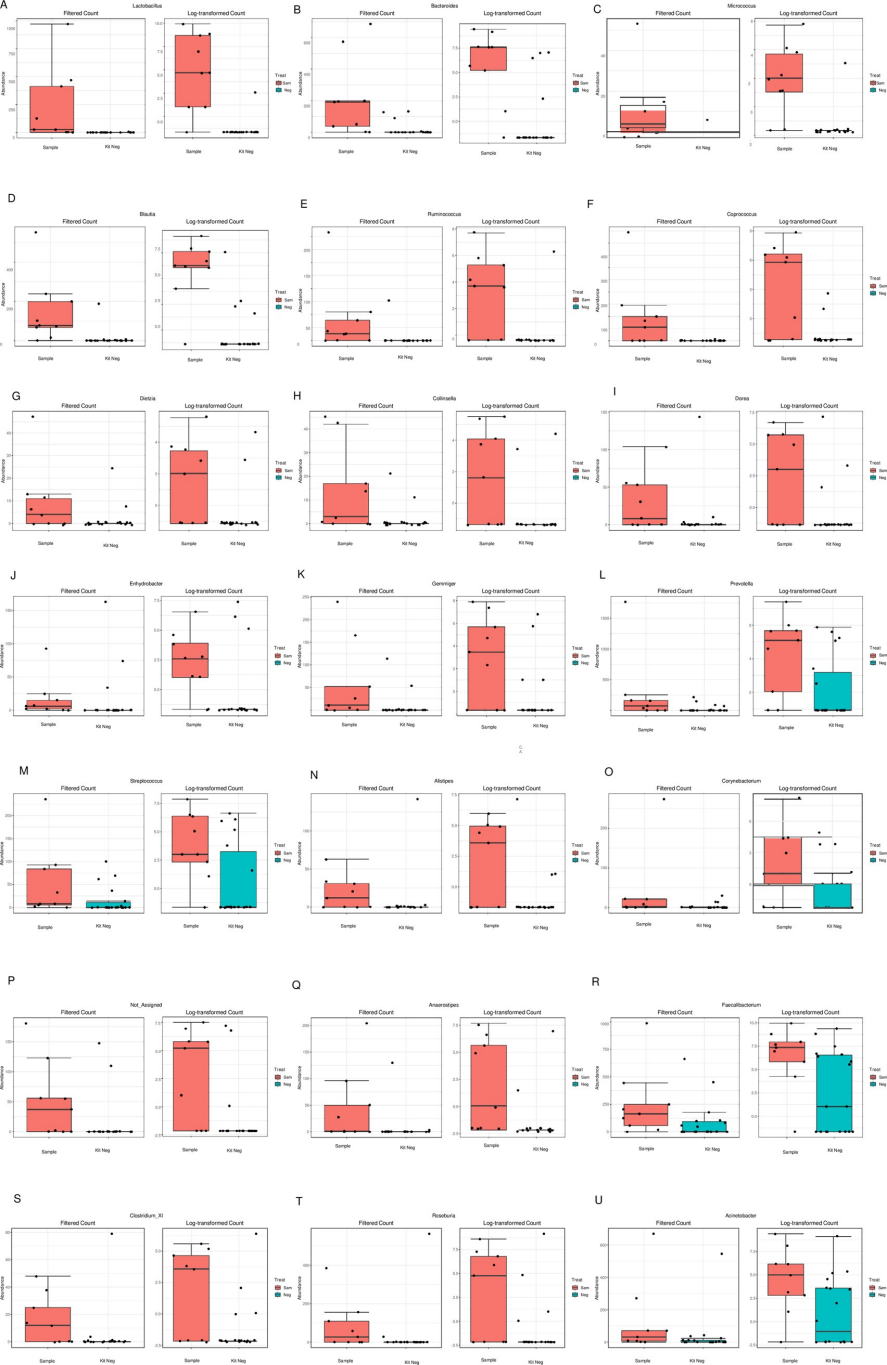
Genera with significantly different read counts between FFPE samples and DNA extraction kit reagent negative controls. Plots created for 21 genera which were detected as significantly greater in Formalin-Fixed Paraffin-Embedded (FFPE) fetal membrane samples compared DNA extraction kit reagent negative controls via univariate analysis. Data presented by read count and log transformed.

**Fig 4:**
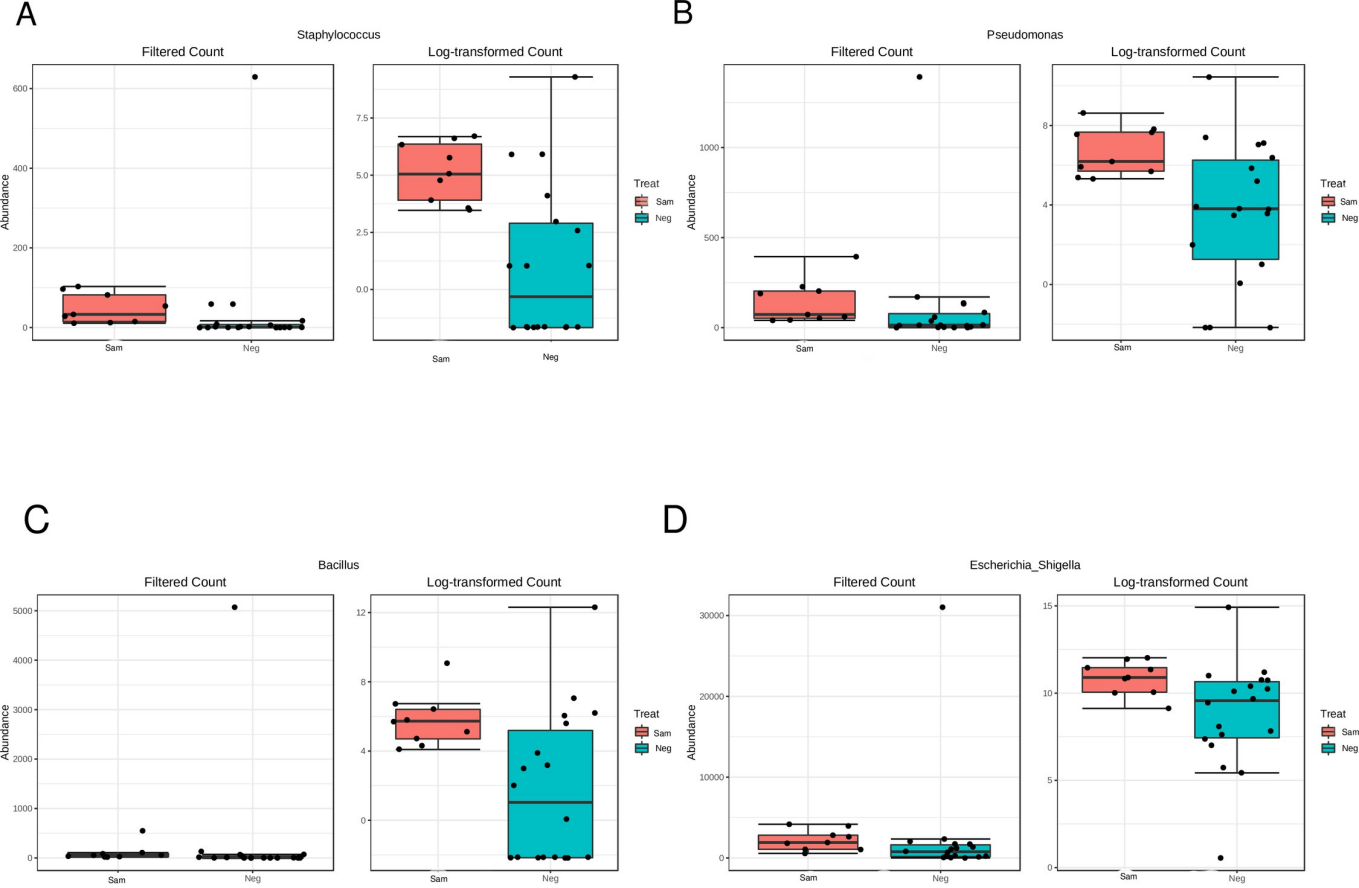
Genera with significantly greater read counts in DNA extraction kit reagent negative controls compared to FFPE samples. Plots created for four genera which were detected as significantly greater in DNA extraction kit reagent negative controls compared to Formalin-Fixed Paraffin-Embedded (FFPE) fetal membrane samples via univariate analysis. Data presented by read count and log transformed.

As detected previously, Escherichia/Shigella was the most abundant genera in samples (36%), also they were the most abundant in paraffin wax controls (42%) and DNA extraction kit regent controls (28%; S2 Fig). There were no significant difference in read counts between FFPE fetal membranes and matched wax negative controls (Table 1), highlighting similar abundance of contributing genera. Alternatively, there were 25 genera with significantly different read counts between FFPE fetal membrane samples and kit negative extraction controls, highlighting the ability to detect distinct differences (Table 1). Those detected as significantly greater in FFPE samples were *Micrococcus*, *Flavobacterium*, *Enhydrobacter*, *Acinetobacter*, *Lactobacillus*, *Bacteroides*, *Blautia*, *Ruminococcus*, *Coprococcus*, *Dietzia*, *Collinsella*, *Dorea*, *Gemmiger*, *Prevotella*, *Streptococcus*, *Alistipes*, *Corynebacterium*, *unassigned bacterium*, *Anaerostipes*, *Faecalibacterium*, *Clostridium_XI* and *Roseburia* (Table 1 and Fig 3). Four genera had a significantly greater read count from kit negative controls. These were *Staphylococcus* (784 vs 435; Fig 4A), *Pseudomonas* (2083 vs 1280; Fig 4B), *Bacillus* (5428 vs 948; Fig 4C), and *Escherichia/Shigella* (45131 vs 19939; Fig 4D).

### Pre- and post-processing for DNA integrity and the detection of contaminating sources

Pre-processing DNA interrogation included NanoDrop, agarose gel electrophoresis and Bioanalyzer. Findings showed that DNA quantity and quality were significantly reduced in extracted DNA from FFPE samples compared to frozen tissue samples, when analysed by agarose gel electrophoresis (200–400 bp vs >10,037 bp; S3 Fig), and NanoDrop (46.7 ng/μl vs 370.5 ng/μl, p = <0.001; S2 Table). NanoDrop quantity profiles ranged from 79.5–975.1 ng/μl in frozen, and 12.6–135.6 ng/μl in FFPE samples. Purity levels were optimum and comparable across preparation types (1.8 vs 1.8, p = 0.940). Bioanalyzer gel results confirmed that FFPE fetal membranes contain highly fragmented DNA, indicated by multiple bands present, plus low quantity of DNA due to the pale intensity of banding (S3 Fig). The mean peak indicated a DNA fragment size of 397 bp, with a smaller peak at 132 bp, confirming the multiple and short fragmentation of FFPE samples (S3 Fig).

Post-processing analysis included applying Decontam and SourceTracker to Illumina amplicon sequencing data. Decontam output determined that 63% of genera generated in DADA2 from FFPE samples were contaminants, whereas 37% were not contaminating signals. Most genera previously detected as significantly greater in FFPE fetal membrane samples compared to negative controls were detected as non-contaminating genera by Decontam (Table 2). *Staphylococcus* and *Escherichia/Shigella* were detected in greater read counts from DNA extraction kit reagent negative controls, however classed as a ‘true’ non-contaminating genus by Decontam. Eight genera were detected as contaminating genera, including *Bacillus*, *Flavobacterium*, *Salmonella*, *Blautia*, *Alistipes*, *Anaerostipes*, *Faecalibacterium and Clostridium_XI* (Table 2).

**Table 2:** Decontam output classified into true or contaminating genera.

Genus	Assigned to True or Contaminant	p. value
*Escherichia/Shigella*	True	0.396
*Bacillus*	Contaminant	<0.001**
*Pseudomonas*	True	0.004*
*Micrococcus*	True	<0.001**
*Flavobacterium*	Contaminant	<0.001**
*Enhydrobacter*	True	0.005*
*Salmonella*	Contaminant	<0.001**
*Acinetobacter*	True	0.003*
*Methylobacterium*	True	<0.001**
*Lactobacillus*	True	0.007*
*Bacteroides*	True	0.013*
*Blautia*	Contaminant	<0.001**
*Ruminococcus*	True	0.002*
*Coprococcus*	True	0.003*
*Dietzia*	True	0.002*
*Staphylococcus*	True	0.203
*Collinsella*	True	<0.001**
*Dorea*	True	0.003*
*Gemmiger*	True	0.004*
*Prevotella*	True	0.005*
*Streptococcus*	True	0.008*
*Alistipes*	Contaminant	<0.001**
*Corynebacterium*	True	<0.001**
*Anaerostipes*	Contaminant	<0.001**
*Faecalibacterium*	Contaminant	<0.001**
*Clostridium_XI*	Contaminant	<0.001**
*Roseburia*	True	0.005*

Genera were assigned to ‘true’ non-contaminating bacteria or assigned as a ‘contaminating’ genera, via ‘*is not contaminant’* script of Decontam. P-value displayed as to a significance of <0.05* or <0.001**.

**Fig 5:**
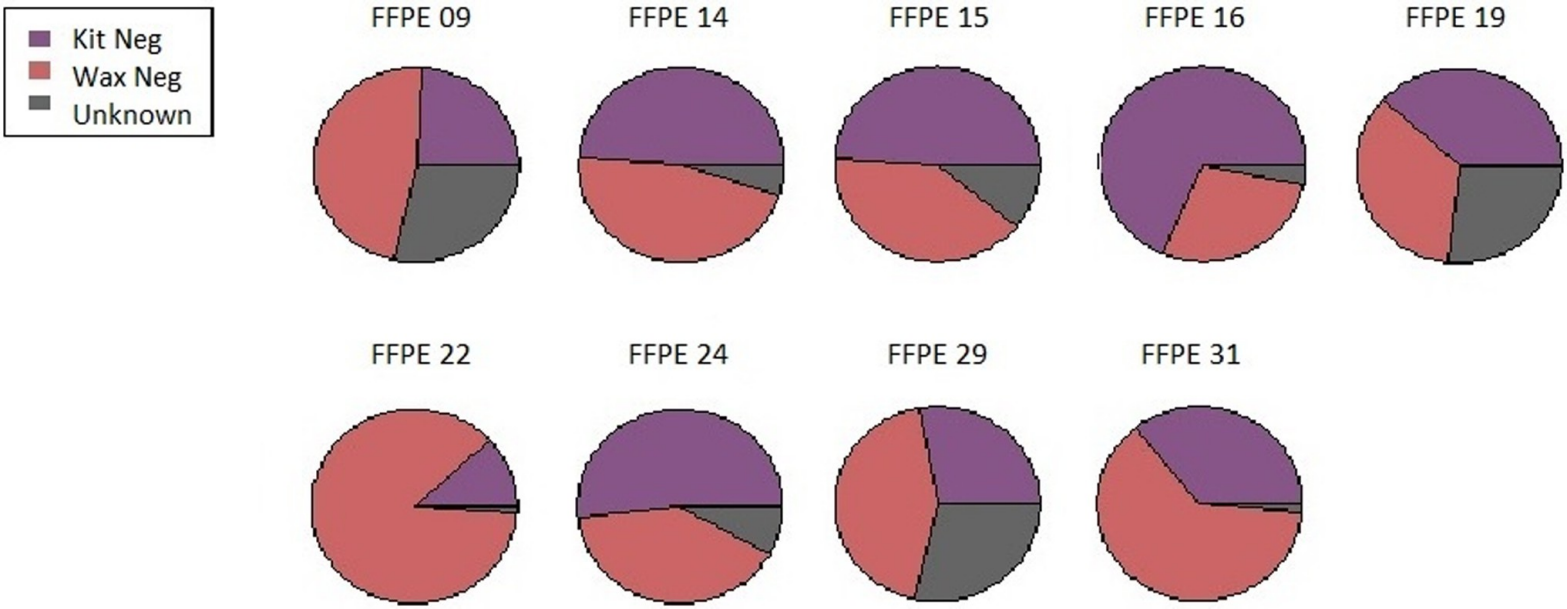
Percentage contribution for bacterial source in Formalin-Fixed Paraffin-Embedded (FFPE) fetal membrane samples. SourceTracker results displayed as a pie chart per individual patient sample. Source origins include DNA extraction reagents (Kit Neg), paraffin wax surrounding the tissue sample (Wax Neg), and source of unknown origin (Unknown).

SourceTracker detected that most of the microbial contribution in FFPE fetal membrane samples may have origins from the surrounding paraffin wax and the DNA extraction kit reagents. An average of 48% of FFPE sample composition was matched to the source of paraffin wax controls (28%-87%; Table 3), with kit reagents being the source of origin for an average of 40% (12%-68%; Table 3). Detection of unknown origin matched to between 1% and 29% (13% average; Table 3), but this was not the greatest contributor to any FFPE samples (Fig 5). Alternatively, unknown sources were the main contributing origin for all frozen samples, with an average contribution of 80% (51%-100%; Table 3). Unknown sources matched 100% as the origin source in three frozen patient samples. Bacteria matched to the source of DNA extraction kit reagents for an average of 20% of frozen samples (0%-49%; Table 3). Yet, this source did not dominate the microbiota in any of the frozen fetal membrane samples.

**Table 3:** Proportion contribution to determine origin source of microbial composition in samples.

Sample Type	Sample ID	Proportion contribution from Wax control	Proportion contribution from Kit control	Proportion contribution from Unknown
**FFPE**	09	47%	24%	29%
	14	46%	49%	5%
	15	40%	49%	11%
	16	28%	68%	4%
	19	35%	38%	27%
	22	87%	12%	1%
	24	40%	52%	8%
	29	43%	28%	29%
	31	63%	36%	1%
**Average**		**48%**	**40%**	**13%**
**Frozen**	09	-	49%	51%
	14	-	29%	71%
	15	-	49%	51%
	16	-	0%	100%
	19	-	10%	90%
	22	-	38%	62%
	24	-	0%	100%
	29	-	8%	92%
	31	-	0%	100%
**Average**		-	**20%**	**80%**

SourceTracker was used to determine the percentage contribution and proportion of contributing bacteria within the sample microbiota originating from the surrounding paraffin wax (Wax control), DNA extraction reagents (Kit control) or unknown source. Findings presented per sample type (Formalin-Fixed Paraffin-Embedded (FFPE) or frozen), patient number and average per group displayed.

## Discussion

The comparison of clinical FFPE and frozen fetal membrane sequencing outcomes have not yet been presented in the literature, with studies focussing on high biomass samples, or artificial scenarios [9,14–16,19]. Thus, the aim of this research was to compare the DNA quality and bacterial profiles of matched paired FFPE and frozen fetal membrane samples, to determine if FFPE membranes can be beneficially used in microbiota research.

Overall, this study shows a difference in bacterial communities and nine individual genera between freezing and fixing identical patient tissues. Finding highlight that the microbiota of FFPE processed fetal membranes is indistinguishable from the surrounding paraffin wax, with no difference in community composition or individual genera. Furthermore, 25 individual genera differed between FFPE samples and DNA extraction reagent kit negative controls, plus over half of bacteria detected in fetal membranes were classified as contaminants when compared to negative controls. The source origin in FFPE fetal membrane samples was mainly attributed to the surrounding paraffin wax structures. This suggests that the processing methods highly influence the microbiota in fetal membrane samples, with the potential of FFPE processing steps to bias the microbiota.

**The microbiota of FFPE and frozen stored fetal membrane samples is not comparable**, highlighting the influence and impact of processing methods on the microbiota of paraffin embedded samples detected following Illumina sequencing.

Clear differential clusters for FFPE and frozen fetal membrane samples were detected prior to Decontam. Duplicate patient samples did not cluster together, thus the microbiota between samples from a given material type tends to be more similar than they are to the microbiota of their matched paired sample taken from the same patient. This highlights the diversity displayed following fixative and embedding processes, especially due to the inability to distinguish between tissue samples and the surrounding paraffin wax from these results. Prior research using colorectal cancer FFPE and frozen specimens also found that paired samples cluster separately, but group by preservation method [16]. Few studies have compared FFPE and frozen fetal membrane tissues in an obstetric setting. One study using qPCR analysis highlighted high variability in amplification efficiency between FFPE and frozen placental tissues, with modified protocols required to achieve successful amplification [38]. In addition, O’Reilly, Barak and Penn (2021) compared the gene expression of known placental reference genes in FFPE and frozen placental discs, successfully employing computation database methodology to show comparable findings [22]. Identical patient samples should present comparable microbiota, if pre- and post-processing of samples has minimal impact on the tissue microbiota. Yet, samples clustered by tissue preservation method, rather than by patient, highlighting the impact of sample processing.

*Staphylococcus* and *Escherichia/Shigella* were significantly greater in DNA extraction kit negative reagents, yet both were categorised as non-contaminating genera by Decontam, although non-significant. *Escherichia/Shigella* was also the most abundant genera across all sample preparation types and negative controls. High detection in all patient samples could be due to high genuine levels in fetal membranes, as *Escherichia* is the suggested main component of the placental microbiota, when compared to other body sites, including oral and vaginal [24]. However, the high abundance in samples and controls questions the origin of these bacteria, and this genus has been the focus of many contamination debates [39], with *Escherichia/Shigella* identified as a primary contaminating bacterium in low biomass microbiota research originating from processing and methodology [18,20,40,41]. *Staphylococcus* have also been previously detected in negative controls [18,41]. However, this is a skin surface originating bacterium, which has been linked to maternal origin and transfer onto fetal material during labour [42,43].

*Ureaplasma* and *Prevotella* also highly contributed to frozen fetal membrane samples. *Ureaplasma* is the most frequently isolated bacteria from fetal membranes of patients with an inflammatory condition of HCA, occurring in 59% and 60% of preterm and term membranes with chorioamnionitis, respectively [44]. *Prevotella* is a Gram-negative commensal oral bacterium, which has been detected in 47% of HCA placenta with bacterial vaginosis via culture techniques [45]. *Prevotella* has been strongly linked to vaginal pathologies and dysbiosis including bacterial vaginosis [46], and negative neonatal outcomes including preterm birth [47]. Such findings highlight the reasoning for detection of both *Ureaplasma* and *Prevotella* here and suggests the representative microbiota of frozen samples.

Nine genera were significantly different between FFPE and frozen fetal membranes, seven of which were greater in FFPE samples. Most genera previously highlighted as greater in FFPE samples were assigned to the true non-contaminating category via Decontam, suggesting sample origin, as decontam functions via prevalence and abundance filtering [21]. Genera highlighted as true from the fetal membrane microbiota may be of maternal origin transferred to the fetal membranes during delivery. These genera such as *Lactobacillus* have previously been detected on fetal material following vaginal delivery and attributed to the vaginal canal as a vaginal commensal bacterium [42,43]. In addition, *Prevotella*, has specifically been linked to preterm patients, as used within this research, or patients with an inflammatory condition, with the detected genera acting as an opportunistic pathogen [29,43,44]. However, the detection of these biologically relevant taxa in negative controls may question their origin.

The genera detected as contaminants by decontam were mainly found in DNA extraction kit controls, with low to no read counts in FFPE samples, this including *Flavobacterium* and *Salmonella*. These genera have previously been identified as originating from DNA extraction kit reagents and elution buffer [17,25,40,41]. These two genera were detected as significantly greater in frozen samples yet presented with low detected read counts. The detection of which has not previously been reported from fetal membrane samples. Applying further methods of research may clarify and confirm these findings, for examples genera or species-specific qPCR. Presence in frozen samples could also be attributed to manufacturer specific DNA reagent origin as these genera were not detected in FFPE samples. Awareness of the bias from different manufacturers is needed when analysing microbiota data, as the variation in reagents from different manufacturers kits could lead to incorrect assumptions about the microbiota of tissue samples [6,17,18,25]. Most research comparing FFPE and frozen samples universally apply specific Qiagen kits to respective samples, resulting in concordance across DNA quality and quantity [48–50]. Using the same kit manufacturer across FFPE and frozen tissue extraction may limit differences due to comparable reagents [40]. Following this recommendation may have been beneficial within our research, however, specific kits were selected here as they were each respectively applied to identical FFPE and frozen samples used in previous research [28,29], allowing comparison and expansion of this research project.

Although significance was detected, univariate plots highlight that the variation in all those genera may be due to the individual patient, rather than the overall cohort, due to the absence of detection in one subset, or the low read counts detected. The higher detected read counts from one or two individual patient samples may have biased the results. However, this highlights patient variation when working with clinical samples. No standard microbiota of the fetal membranes have been clarified, and debate continues [14,51,52]. This reduces the ability to detect which genera are potential contaminants or understand what a diseased state microbiota would entail, as no healthy baseline for comparison is present. The diversity identified between samples seen here may contribute to explaining the inability to clarify a standard fetal membrane microbiota. Patient variation enhances the importance of large sample subsets. The low patient numbers included here (n = 9) may have reduced the representation and reflection of a wider cohort.

The differences detected between FFPE and frozen samples could also be due to the reduced quality and quantity of DNA extracted from FFPE fetal membranes, as DNA fragmentation and cross-linkage may decrease the success of downstream methodologies [5,53]. FFPE DNA was highly fragmented as shown by agarose gel electrophoresis and Bioanalyzer, with reduced quantity detected by NanoDrop. The quality of DNA extracted from FFPE samples was suboptimum, with fragmentation detected at 200–400 bp. Fragmentation levels of 500 bp have been previously detected in FFPE placental tissues [38]. Fragmentation occurs due to the fixative process introducing cross-linking between amino acids, leading to DNA degradation which may negatively impact DNA extractions [5,9,10,53]. Current commercial DNA extraction kits used in this research aim to limit the issue of cross-linkage by modified methodologies, including chaotropic salt-based lysis combined with increased incubation temperatures [54,55]. However full eradication of pre-processing fixative effects is difficult. An alternative fixative reagent which bridges the gap between FFPE and frozen processing methods may improve clinical comparison and application of microbiota research. PAXgene tissue fixative reagents have been suggested as this intermediate method [56]. This uses a reduced four-hour fixative time compared to 24 hours in formalin [56]. The aim of PAXgene is to preserve sample morphology yet decrease fragmentation, leading to increased template size for qPCR and sequencing [56]. However, longitudinal studies are required to understand long term preservation following this method. The short fragment lengths created by sample processing may restrict target primer size in qPCR and sequencing methods [48,57]. Variable success in primer binding levels have been detected as target gene length is increased in qRT-PCR of FFPE placental disc samples, suggesting lower quality extracts from FFPE samples [22]. In our research here, the short 250 bp gene fragment for the V4 16S rRNA gene region was targeted, aiming to generate optimum results from fragmented DNA of FFPE samples, and to avoid amplification bias [58]. Alternatively, further modification of protocols may be required to enhance the beneficial use of FFPE samples, including decreasing the target fragment to 50–75 bp in Illumina sequencing [12,14], or sequencing multiple variable regions of the 16S rRNA gene to provide greater representation [59]. Fragmentation in FFPE samples has also been linked to processing methods and storage durations [12]. Samples used in this study were stored for a maximum of three years, with a three-to-seven-year limit for optimum downstream results [5,13]. Degradation status analysed via a longitudinal study, covering zero to ten years, would be beneficial to optimise storage protocols if FFPE samples were to be included in routine microbiota research.

### Microbial contribution detected from external sources

SourceTracker displayed high level of externally originating bacterial contribution to the FFPE samples. This found to be on average a half from the surrounding paraffin wax block and 40% from the DNA extraction kit reagents. This highlights the influence of contamination from these sources on FFPE samples. The low biomass sample enhances the potential for processing contaminates to obscure the tissue originating signal, as only 13% of bacteria was attributed to sources other than the negative controls. The high contribution from external sources suggests a low microbial biomass originating from samples. This could be due to the inability to extract DNA using the methods selected, the low quality and quantity of DNA that was extracted or the fragmented DNA impacting primer binding, leading to lower detection sensitivity and resolution, as discussed previously [16,53].

There was an inability to differentiate the read counts of individual genera between FFPE and wax negative controls as none of the genera detected were significantly different between FFPE fetal membrane samples and paraffin wax negative controls. This combined with the high percentage of bacteria from FFPE samples matched to the origin source of paraffin wax via SourceTracker suggests the inability to detect a microbiota purely originating from FFPE fetal membrane samples. In this case the infiltrating wax microbiota may modify and or mask the detection. Previous research has detected comparable genera and overlapping clusters in colorectal FFPE samples and wax negative controls, yet comparison between specific genera were not discussed [16]. The same was found when comparing FFPE samples and DNA extraction kit negative controls, but the distinction was that tissues and kit negatives clustered separately [16]. Alternatively, protein composition remains following paraffin fixation as most proteins studied overlap in both FFPE processing and snap freezing colon samples [9]. The high contribution from paraffin wax to FFPE samples could also be due to the inability to fully segregate the tissue microbiota from the wax microbiota when sectioning the FFPE samples–thus residual wax microbiota could remain within the sample, or vice versa. This could then be carried over into microbiota analysis and identified as contributing to the sample microbiota. The direction of bacteria translocation from tissue to paraffin wax or the reverse direction cannot be easily determined. During processing of FFPE samples the tissue is submerged, embedded, and penetrated by the paraffin wax, which replaces all water content within tissue samples [56,60]. Thus, bacteria identified as non-contaminating genera in samples may have originated from other sources including the preparation of the fixatives or the environmental microbiota of the laboratory carrying out the protocol, which was not assessed or included as a reference. The comparison of samples to paraffin wax surrounding respective tissues as performed here is beneficial, however it is difficult to elucidate the origin of bacteria due to the close interaction of the tissue and the infiltrating wax. Laser capture dissection to retrieve samples from the surrounding wax before processing may increase the accuracy of separation and decrease the detection of remnant wax contribution [61]. Inclusion of an empty blank paraffin wax block prepared at the time of the samples and processed following the identical procedure would act as a control and would be beneficial to further investigate the paraffin wax processing microbiota [27]. In addition, the use of a wax biological standard for FFPE samples, for example, Protoblock could also function as a quality control system [53]. This bacterial cell matrix represents FFPE samples and is to be processed alongside tissues to assess protocols and further identify and confirm contamination arising from methodology [16,53].

Continuing with SourceTracker, all frozen samples had the highest microbial contribution from unknown sources, suggesting that contamination from kit reagents was reduced in these sample types. This possibly due to a higher level of microbial load or biomass, which could be confirmed by bacterial load analysis via 16S rRNA targeted qPCR. This has previously been detected with increased PCR product in normal and tumorous frozen samples compared to FFPE tissues from multiple body sites [62]. Two frozen samples presented with 100% unknown bacterial origin. Unidentified origins are sources not supplied to the training reference set, as those sources included were only paraffin wax and DNA extraction kit reagents. Thus, the unknown bacterial origin could include the fetal membrane tissue sample microbiota source. However, with no positive control for comparison, these sources could also be additional environmental or body site locations, as discussed previously. Inclusion of vaginally sourced samples could have been beneficial for confirming the origin of an unidentified source, as previous studies have tracked the source of origin on placental and obstetric tissues to vaginal and/or oral locations [63,64]. However, these studies did not compare to maternal skin, which may also be a potential source of contamination during delivery [39,65]. SourceTracker therefore highlights the reduced contribution of contamination to frozen samples, but greater bias to detecting contamination on FFPE samples over the tissue sample signal.

### Study strengths and limitations

Specific procedures were following during this study to reduce and identify contaminating sources in low biomass fetal membrane samples. No standardised sample protocol has been published for the preparation and use of FFPE specimens in microbiome analysis, thus the use of different protocols across medical and research centres may lead to variation in results and bias analysis [20]. During sequencing a positive control of mixed microbial communities was used to assess bias and provide accurate taxonomic identification by standardised methods. However, the controls of bacterial serial dilutions or positive spiked titrations could have been used to detect cross-contamination and to understand bias [20]. Minimal positive controls were used to clarify the limit of detection for this methodology. Alongside environmental contamination, human mitochondrial DNA can act as an additional source of contamination, with host DNA outnumbering target bacterial DNA in 16S rRNA sequencing [66–68]. The use of a microbiome enrichment DNA extraction kit may be beneficial for FFPE samples [67], with the inclusion of a DNA repair step requiring further validation, as suggested previously [68]. Additional repeat experiments could include the use of propidium monoazide (PMA) to reduce human DNA amplification, reduce bias, and inhibit amplification of nonviable non metabolically active bacteria [53,68–70].

Negative controls used here included DNA extraction kit reagent negative controls for frozen tissues and the addition of surrounding paraffin wax block controls for the FFPE prepared samples. Along with including these negative controls within the methods, the results of which were analysed by two different computational analysis packages, to identify the impact they have on the sample microbiota. Comparable studies also analysed additional negative controls, including sterile air swabs from the clinical laboratory, sterile unopened swabs, plus additional body site samples for environmental and human contamination controls [25]. This could lead to improved detection and category assignment when using decontam, plus the ability to greater detect and understand the origin of any contamination via SourceTracker. The inclusion of one of each negative control type per batch to monitor batch effects and cross contamination would be beneficial [20]. Including additional body site samples may detect genera of greater clinical relevance, however, the inclusion of a wax negative control in our study better reflects variation in sample preparation, which was the aim of the research conducted.

The quantity and quality of extracted DNA was monitored prior to sequencing as understanding DNA quantity, quality and fragmentation is crucial to determine if the assigned target sequence will amplify. Combining multiple analysis methods, as used here improves the accuracy and reliability of findings. However, the NanoDrop has been suggested to overestimate DNA quantity, and the reliance of single stranded DNA analysis limits detection [49]. Although additional methods were used to assess DNA fragmentation, including agarose gel electrophoresis and Bioanalyzer. The use of a Qubit periodically throughout sample preparation may be more beneficial for accurate detection of DNA quality and quantity in low biomass materials [71].

Matched paired patient analysis strengthens these findings, However, the inclusion criteria of only patients with both FFPE and frozen membrane samples collected and prepared led to the analysis of a small patient subset (n = 9). FFPE samples were processed as one combined amniochorionic fetal membrane roll, whereas each amnion and chorion membrane was processed individually in frozen preparations. Computationally combining the amnion and chorion membranes from frozen samples aimed to better reflect the combined FFPE fetal membrane rolls.

This research has aimed to control for, describe methods applied to, or confront variables, to meet the RIDE minimum guidelines for low microbial biomass studies [20], but additional factors may also impact sample composition. This could include sample processing methods, storage time, DNA preparation method, DNA damage, the methods used for DNA integrity and DNA purification, plus the hypervariable target region used [13,16,20,53]. The methodological strategies presented in this research may not be suitable for analysis and comparison of the sample material studied, plus the use of only one lab based microbial method may limit the reliability of findings. Further inclusion of additional methodology would be beneficial to clarify, confirm and support the sequencing data presented here, including bacterial load qPCR analysis to accurately measure bacterial abundance in low biomass samples compared to wax controls and kit reagent negative controls [20]. Multiple post-processing computational methods were applied to data analysis, including Decontam and SourceTracker. These methods are recommended rather than simply removing all genera present in negative controls [72]. Decontam has been beneficially applied to placental research previously [21,25], where <1% of ASVs were assigned to the true non-contaminating category, compared to 37% assigned to the non-contaminating category within our fetal membrane research displayed here. In the Davis *et al* (2018) and Lauder *et al* (2016) study, all samples were taken from term patients [21,25], whereas we specifically selected for the use of preterm patients here in our study. This was due to our previous findings indicating that preterm patients have increased bacterial presence, with decreased probability of contaminating bacteria dominating over the tissue microbiota [29]. Decontam strictly assigns a bacterium to ‘genuine/true’ or ‘contaminant’ category, without the option of being both. This distinctive classification may not be representative of the clinical environment and bacteria of human origin. Also, a change in read count by just one number can impact the classification bracket which the bacterium is assigned to [21]. Data were therefore also analysed via SourceTracker, to improve detection and distinguish the origin of bacteria from samples and controls [73]. In addition to Decontam and Source Tracker, the FAST microbial source tracking pipeline could have also been employed for increased accuracy [74].

These findings have aided in understanding the benefits and pitfalls of clinical sample collection and storage methods; to educate, improve and enhance current practice and applications in a clinical and research setting. If FFPE fetal membrane samples are to be used in microbiota research, then further optimisation of current protocols is required. This may include standardising tissue preparation methods, fixative times and providing a limit to storage duration. The inclusion of pre- and post-reduction of contamination must also be considered.

## Conclusion

The use of FFPE samples in microbiota research would reduce the time, cost, resource requirements, processing steps and storage limitations currently present in frozen tissue processing. This research finding is one of the first to compare FFPE fetal membranes to the gold standard frozen tissues used in microbiota research. Findings here show that FFPE and frozen fetal membranes are not of comparable high quality required for microbiota analysis, as the influence of paraffin wax may lead to fragmentation of DNA and the addition of contaminating bacteria, both of which may bias the microbiota detected using the sequencing methodology here. This research highlights the requirement for a standard and universal method for the preparation, processing, storage, research methods and analysis of FFPE fetal membrane samples, and this research has suggested ways in which to improve current methodology and recommendation for future studies.

## Supporting information

S1 Fig Relative abundance of matched paired fetal membranes. Relative abundance plots from V4 16S rRNA amplicon sequencing of extracted DNA from matched paired fetal membrane samples divided by tissue preparation type of Formalin-Fixed Paraffin-Embedded (FFPE) or frozen tissue samples.
(TIF)

S2 Fig Relative abundance of FFPE fetal membranes and negative controls. Relative abundance plots from V4 16S rRNA amplicon sequencing of extracted DNA from Formalin-Fixed Paraffin-Embedded (FFPE) fetal membrane samples, plus negative controls of surrounding corresponding paraffin wax (WAXN) and DNA extraction kit reagent negative control (KNEG).
(TIF)

S3 Fig DNA quality and quantity analysis via Agarose gel electrophoresis and Bioanalyzer from fetal membrane samples. Agarose gel electrophoresis image of a subset of DNA extracts from matched paired fetal membranes of either frozen membrane samples (A), or Formalin-Fixed Paraffin-Embedded (FFPE) samples (B) performed via agarose gel electrophoresis and imaged via GBox SynGene Chemi-XX6. Lane one and lane 14 represent 1KB reference HyperLadder. Example of a computational electrophoresis image from a subset of Formalin-Fixed Paraffin-Embedded (FFPE) samples (C), plus sample electropherogram (D) and ladder electropherogram (E) from Bioanalyzer Agilent 2100. Ladder is included (L), with upper (purple) and lower (green) markers visible across samples (lane 1–11).
(TIF)

S1 Table Matched paired patient demographics. Patient characteristic data from patients providing fetal membrane samples for the study, assessed from matched paired Formalin-Fixed Paraffin-Embedded (FFPE) and frozen fetal membranes. Unless stated data is displayed as n (%).
(DOCX)

S2 Table Example of NanoDrop spectrophotometer sample results following DNA extractions. NanoDrop results from each patient sample in triplicate from the formalin-fixed paraffin-embedded fetal membrane rolls (FFPE) or matched paired frozen tissue sample labelled by patient identification number. DNA concentration (ng/μl), plus purity ratio (A260/280, A260/230) displayed.
(DOCX)

S1 File Keywords, conflicts of interests, details of ethical approval, funding information and word count. (DOCX)

S2 File (ZIP)

S1 Data TAXA table, metadata and ASV count data provided from Illumina sequencing results and following processing through DADA2 and Bioconductor. (XLSX)

S1 Dataset Raw FASTQ files compressed into a .zip folder. (XLSX)

S2 Dataset (ZIP)

S1 Protocols Genomic DNA extraction, DNA interrogation and Illumina amplicon sequencing protocols. Protocols for Formalin-Fixed and Paraffin-Embedded (FFPE) fetal membrane DNA extraction from BiOstic DNA extraction kits and frozen sample DNA extraction from Qiagen DNA extraction kits. DNA quality and quantity assessment protocols including NanoDrop, agarose gel electrophoresis and Bioanalyzer. Additional protocol specifics for the amplification of the 16S rRNA V4 target region via MiSeq.
(DOCX)
