# Supplementary figures and images for: Formalin-Fixed Paraffin-Embedded (FFPE) samples are not a beneficial replacement for frozen tissues in fetal membrane microbiota research
Source: PLoS One. 2022 Mar 17;17(3):e0265441. doi: 10.1371/journal.pone.0265441 (PMC8929612; doi:10.1371/journal.pone.0265441)

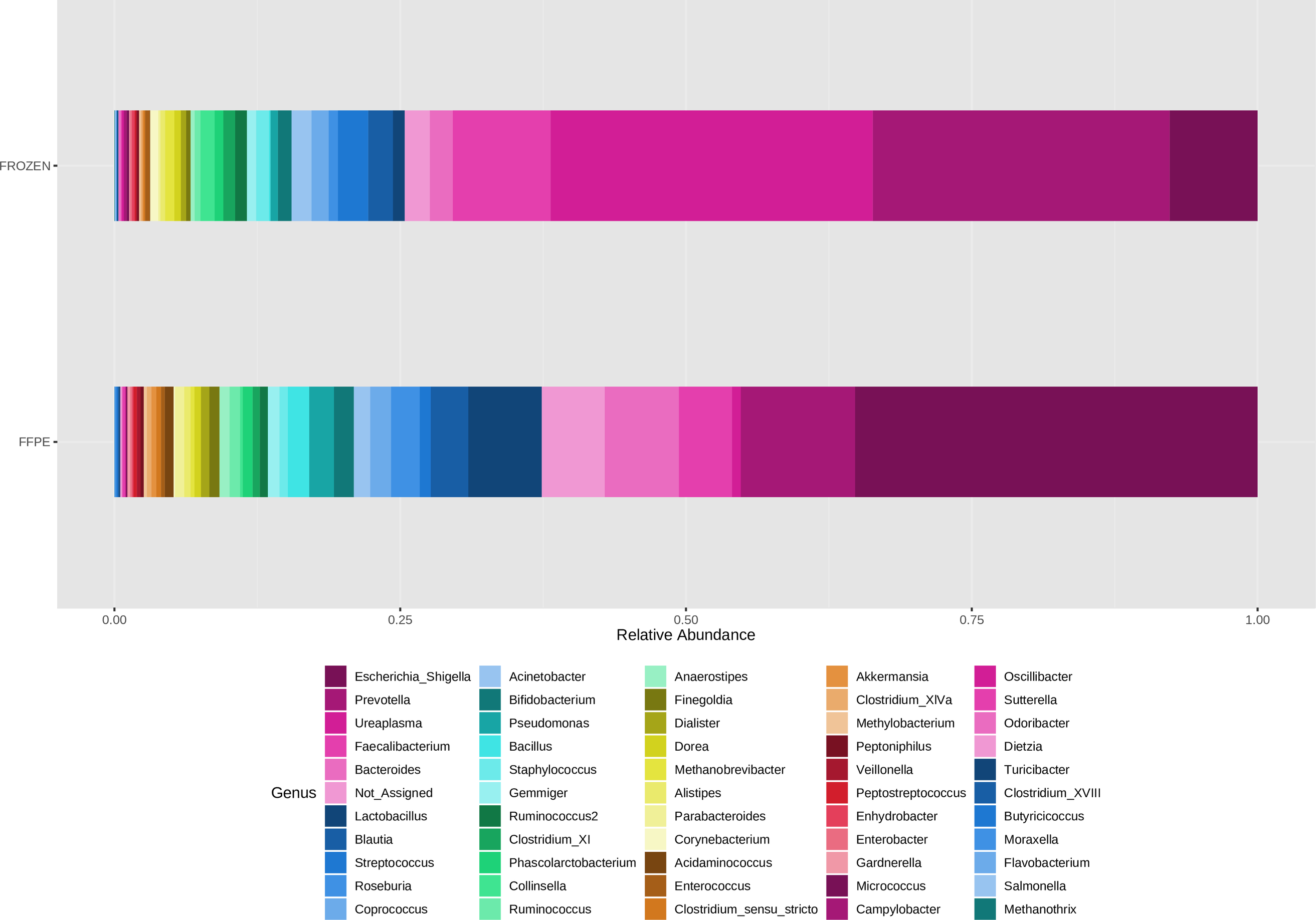

Supplement: S1 Fig — Relative abundance plots from V4 16S rRNA amplicon sequencing of extracted DNA from matched paired fetal membrane samples divided by tissue preparation type of Formalin-Fixed Paraffin-Embedded (FFPE) or frozen tissue samples. (TIF) [file pone.0265441.s001.tif]

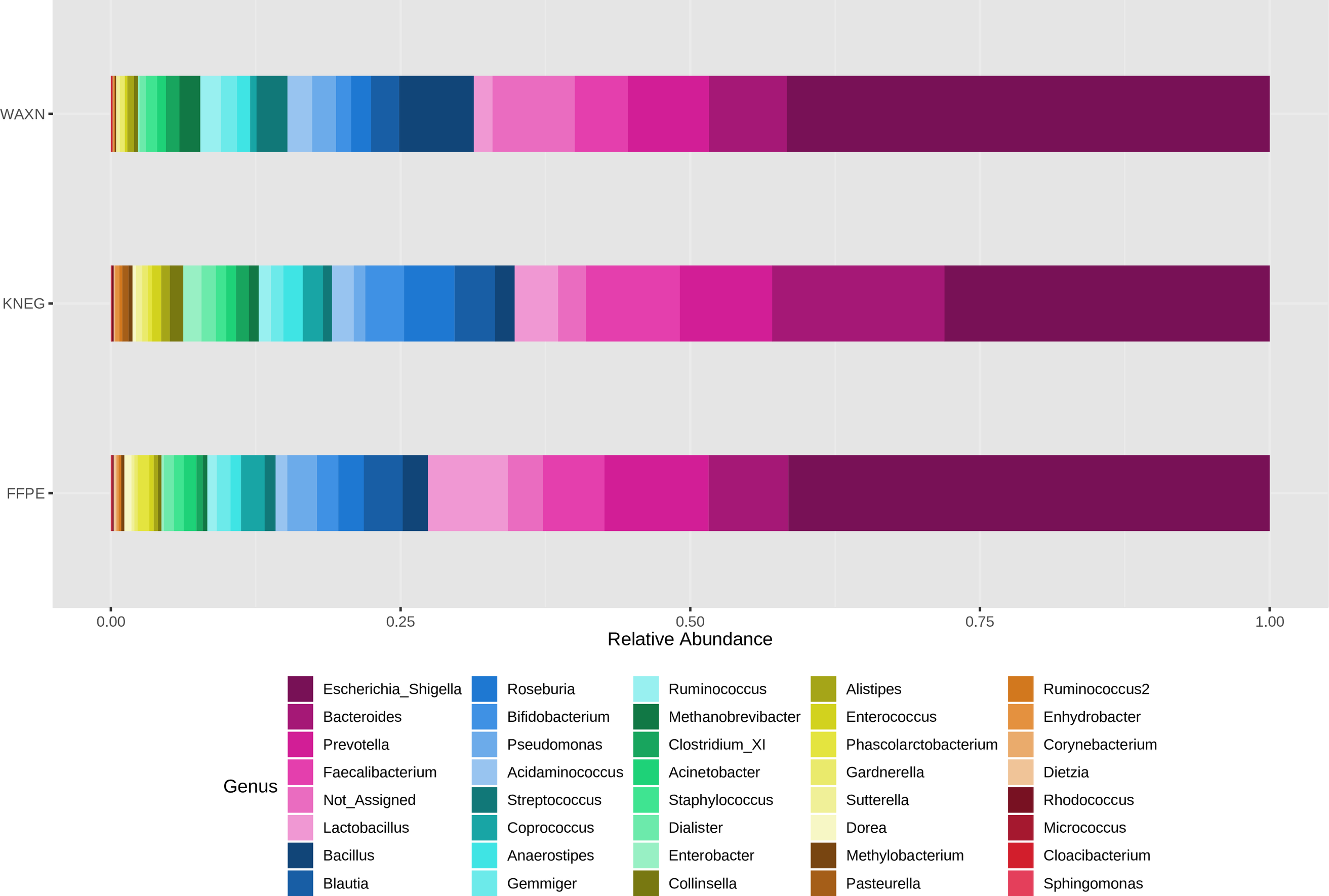

Supplement: S2 Fig — Relative abundance plots from V4 16S rRNA amplicon sequencing of extracted DNA from Formalin-Fixed Paraffin-Embedded (FFPE) fetal membrane samples, plus negative controls of surrounding corresponding paraffin wax (WAXN) and DNA extraction kit reagent negative control (KNEG). (TIF) [file pone.0265441.s002.tif]

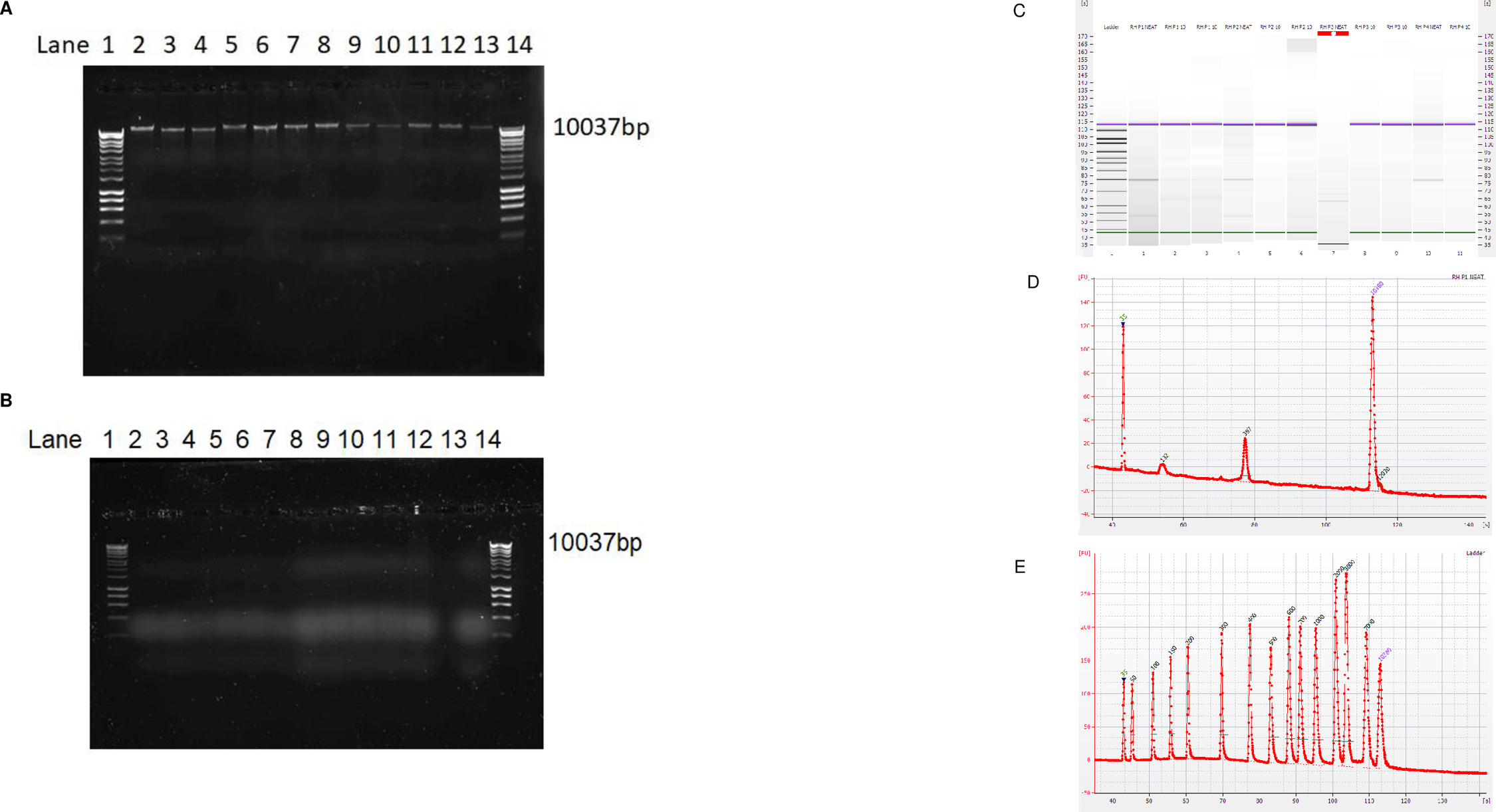

Supplement: S3 Fig — Agarose gel electrophoresis image of a subset of DNA extracts from matched paired fetal membranes of either frozen membrane samples (A), or Formalin-Fixed Paraffin-Embedded (FFPE) samples (B) performed via agarose gel electrophoresis and imaged via GBox SynGene Chemi-XX6. Lane one and lane 14 represent 1KB reference HyperLadder. Example of a computational electrophoresis image from a subset of Formalin-Fixed Paraffin-Embedded (FFPE) samples (C), plus sample electropherogram (D) and ladder electropherogram (E) from Bioanalyzer Agilent 2100. Ladder is included (L), with upper (purple) and lower (green) markers visible across samples (lane 1–11). (TIF) [file pone.0265441.s003.tif]
