# Supplementary material for: Formalin-Fixed Paraffin-Embedded (FFPE) samples are not a beneficial replacement for frozen tissues in fetal membrane microbiota research
Source: PLoS One. 2022 Mar 17;17(3):e0265441. doi: 10.1371/journal.pone.0265441 (PMC8929612; doi:10.1371/journal.pone.0265441)
Supplement: S1 Table — Patient characteristic data from patients providing fetal membrane samples for the study, assessed from matched paired Formalin-Fixed Paraffin-Embedded (FFPE) and frozen fetal membranes. Unless stated data is displayed as n (%). (DOCX) [file pone.0265441.s004.docx]

**S1 Table Matched paired patient demographics.**

|  | **Patients (n=9)** |
| --- | --- |
| Gestational age, weeks (mean (SD)) | 30.1 (2.2) |
| Birthweight, g (mean (SD)) | 1568.9 (437.9) |
| Maternal age, years (mean (SD)) | 26.6 (6.7) |
| Smoker - Yes | 3.0 (33.3) |
| Smoker - No | 4.0 (44.4) |
| Smoker - NA | 2.0 (22.2) |
| Parity, N (mean (SD)) | 1.3 (1.8) |
| Fetal sex - Male | 2.0 (22.2) |
| Fetal sex - Female | 7.0 (77.8) |
| Mode of delivery - Vaginal | 5.0 (55.6) |
| Mode of delivery - Caesarean section | 4.0 (44.4) |

Patient characteristic data from patients providing fetal membrane samples for the study, assessed from matched paired Formalin-Fixed Paraffin-Embedded (FFPE) and frozen fetal membranes. Unless stated data is displayed as n (%).
