# Supplementary material for: Formalin-Fixed Paraffin-Embedded (FFPE) samples are not a beneficial replacement for frozen tissues in fetal membrane microbiota research
Source: PLoS One. 2022 Mar 17;17(3):e0265441. doi: 10.1371/journal.pone.0265441 (PMC8929612; doi:10.1371/journal.pone.0265441)
Supplement: S2 Table — NanoDrop results from each patient sample in triplicate from the formalin-fixed paraffin-embedded fetal membrane rolls (FFPE) or matched paired frozen tissue sample labelled by patient identification number. DNA concentration (ng/μl), plus purity ratio (A260/280, A260/230) displayed. (DOCX) [file pone.0265441.s005.docx]

**S2 Table NanoDrop spectrophotometer sample results following DNA extractions.**

| **Sample Type** | **Sample ID** | **Concentration (ng/µl)** | **A260:A280 purity ratio** |
| --- | --- | --- | --- |
| **FFPE** | 09 | 40.667 | 1.673 |
|  | 14 | 47.913 | 1.943 |
|  | 15 | 39.398 | 1.787 |
|  | 16 | 135.622 | 1.935 |
|  | 19 | 42.387 | 1.983 |
|  | 22 | 36.643 | 1.903 |
|  | 24 | 12.633 | 1.635 |
|  | 29 | 35.753 | 1.835 |
|  | 31 | 29.627 | 1.807 |
| **Frozen** | 09 | 233.140 | 1.870 |
|  | 14 | 975.050 | 1.860 |
|  | 15 | 495.110 | 1.870 |
|  | 16 | 94.040 | 1.870 |
|  | 19 | 360.540 | 1.824 |
|  | 22 | 396.715 | 1.816 |
|  | 24 | 615.532 | 1.846 |
|  | 29 | 84.847 | 1.820 |
|  | 31 | 79.490 | 1.870 |

NanoDrop results from each patient sample in triplicate from the formalin-fixed paraffin-embedded fetal membrane rolls (FFPE) or matched paired frozen tissue sample labelled by patient identification number. DNA concentration (ng/µl), plus purity ratio (A260/280, A260/230) displayed.
