# Supplementary material for: Formalin-Fixed Paraffin-Embedded (FFPE) samples are not a beneficial replacement for frozen tissues in fetal membrane microbiota research
Source: PLoS One. 2022 Mar 17;17(3):e0265441. doi: 10.1371/journal.pone.0265441 (PMC8929612; doi:10.1371/journal.pone.0265441)
Supplement: S1 File — (DOCX) [file pone.0265441.s006.docx]

**S1 File Supporting information**

**Keywords**: Formalin-Fixed Paraffin-Embedded, FFPE, fetal membrane, microbiota, decontam, sequencing.

**Conflicts of Interest: The author(s) declare that there are no conflicts of interest.**

**Details of ethical approval**: The samples were utilised for current research via a transfer agreement, with prior approval from Newcastle and North Tyneside 1 Research Ethics Committee (Ref:10/H0906/71).

**Funding information**: This work was funded by a grant from the British Maternal and Fetal Medicine Society awarded to Dr Waring and Dr Nelson and supported by the Teesside University Graduate Tutor Scheme with resources provided by the School of Health and Life Sciences.

**Word count**

Abstract: 300

Manuscript: 6309
