# Supplementary material for: Formalin-Fixed Paraffin-Embedded (FFPE) samples are not a beneficial replacement for frozen tissues in fetal membrane microbiota research
Source: PLoS One. 2022 Mar 17;17(3):e0265441. doi: 10.1371/journal.pone.0265441 (PMC8929612; doi:10.1371/journal.pone.0265441)
Supplement: S1 Protocols — Protocols for Formalin-Fixed and Paraffin-Embedded (FFPE) fetal membrane DNA extraction from BiOstic DNA extraction kits and frozen sample DNA extraction from Qiagen DNA extraction kits. DNA quality and quantity assessment protocols including NanoDrop, agarose gel electrophoresis and Bioanalyzer. Additional protocol specifics for the amplification of the 16S rRNA V4 target region via MiSeq. (DOCX) [file pone.0265441.s011.docx]

**S1 Protocols Genomic DNA extraction, DNA interrogation and Illumina amplicon sequencing protocols.** Protocols for Formalin-Fixed and Paraffin-Embedded (FFPE) fetal membrane DNA extraction from BiOstic DNA extraction kits and frozen sample DNA extraction from Qiagen DNA extraction kits. DNA quality and quantity assessment protocols including NanoDrop, agarose gel electrophoresis and Bioanalyzer**.** Additional protocol specifics for the amplification of the 16S rRNA V4 target region via MiSeq.

**Genomic DNA extraction of frozen stored fetal membranes**

Total genomic DNA was extracted from 25 mg frozen membranes on ice under sterile conditions via QIAamp Fast DNA tissue kit (Qiagen). Sectioned and weighed fetal membrane tissues were added into individual QIAamp tissue disruption tubes containing a metal bead for mechanical lysis, alongside enzymatic and chemical lysis provided by the reagents. 265 µl of freshly prepared mastermix was added to all tubes, consisting of 200 µl AVE (0.04% NoN_3_ sodium azide), 40 µL VXL (guanidinium chloride and Triton X-100), 1 µl DX reagent (antifoaming buffer), 20 µl Proteinase K and 4 µl RNase A (100 mg/ml). Tubes were vortexed for five minutes in a Vortex-Genie (Scientific Industries) before transferring to a thermal mixer (ThermoMixer, Eppendorf) for ten minutes at 56 °C and 1000 rpm to homogenise samples. To all tubes, 265 µl MVL (guanidine and isopropanol) was added and vortexed for ten seconds, before transferring the solution to QIAamp mini spin columns, with a silica membrane to isolate DNA. Tubes were centrifuged at 13,000 xg for one minute (Microfuge, Sigma), before transferring the spin column into a sterile tube. AW1 buffer (500 µl) was added to all tubes and centrifuged as previous, before transferring the spin column into a sterile tube and repeating with 500 µl AW2 buffer. AW buffers are wash buffers to improve the purity of the eluted DNA without affecting DNA quantity. Once transferred to sterile collection tubes, 50 µl elution buffer (ATE) was added to the membranes to elute nucleic acids. Tubes were incubated for two minutes at RT before centrifuging at 13,000 xg for one minute. Silica membranes were discarded, and the eluted nucleic acids were stored at -20 °C until required for analysis (maximum duration of three months). Kit negative controls were also processed identical to samples, with dH_2_O replacing tissue samples (n=9).

**Genomic DNA extraction of Formalin-Fixed Paraffin-Embedded (FFPE) fetal membranes**

Nucleic acid extraction from FFPE samples were performed via BiOstic FFPE Tissue DNA isolation kit (MoBio) at RT under sterile conditions. Samples were sectioned to 10 µm in triplicate using a manual rotary microtome (Leica, RM2125). External slices were discarded to minimise the impact of environmental contamination. Processed slices were weighed and trimmed to 25 mg and added to collection tubes. 180 µl FP1 and 20 µl FP2 deparaffinisation solutions were immediately added to each tube to chemically and enzymatically dissolve the wax. Tubes were vortexed on a Vortex-Genie for 20 seconds and centrifuged (Microfuge, Sigma) at 13,000 xg for 30 seconds to bring the wax into contact with the buffers. 20 µl FP3 was added to the solution and vortexed for ten seconds before incubating in a heat block at 55 °C for two-hours. This was followed by a one-hour incubation at 90 °C. FP3 contains Proteinase K which enhances the breakdown of DNA-protein cross-linkage, with optimum performance at 55 °C. Following incubation, tubes were centrifuged at 13,000 xg for one minute and the digested solutions were transferred to new collection tubes, avoiding the transfer of remaining wax segments. 200 µl of FP4 was added to tubes and vortexed for ten seconds. This chaotropic salt buffer aids DNA binding to silica membranes. 200 µl 100% ethanol (FP5) was added to the tubes and vortexed for ten seconds to improve binding conditions. Extracts (650 µl) were transferred onto silica membrane spin columns used to purify and isolate DNA. Spin columns were centrifuged at 10,000 xg for one minute and excess solution discarded. 500 µl wash buffer (FP6) was added directly onto the membrane and centrifuged at 10,000 xg for one minute to remove excess proteins and contaminants. The filter column was transferred to a new sterile tube before adding 500 µl of ethanol wash solution (FP7) and centrifuging as previously. Excess solution was discarded, and tubes were centrifuged at 13,000 xg for two minutes before transferring the silica membrane to a new sterile tube. To the dried filter membrane, 50 µl elution buffer (FP8) was added and incubated at RT for five minutes. Tubes were centrifuged at 10,000 xg for one minute. The spin column was discarded, and eluted DNA stored at -20 °C until required for downstream processing (maximum duration of three months). Wax negative controls formed of excess paraffin wax which surround corresponding FFPE fetal membrane tissues were analysed alongside samples to identify the contribution of microorganisms within the wax. DNA extraction kit negative controls (n=18) were processed alongside with identical techniques**.**

**NanoDrop spectrophotometer protocol**

To confirm successful DNA extraction and monitor yield and purity of DNA, NanoDrop spectrophotometer (1000, V3.8.1; Thermo Fisher) was performed on extracted DNA from FFPE and frozen fetal membrane tissues, plus wax and kit negative controls. The system was cleaned using1 µl dH_2_O and calibrated using 1 µl of respective elution buffer which the DNA is stored in, before measuring samples (1 µl) in triplicate. DNA concentration (ng/µl), and purity (A260:280 and A260:230) were assessed. Average concentration values were calculated and used for sequencing preparation.

**Agarose gel electrophoresis protocol**

A 1% (w/v) agarose gel electrophoresis was performed to further assess DNA quality. Agarose was dissolved in 1X TBE with 0.1 µl/ml SYBR Safe DNA gel stain (Invitrogen) and set in agarose gel tanks. 10 µl of DNA product of interest was combined with 4 µl DNA loading dye (Bioline, 5X) and 6 µl of this loaded into corresponding wells of the agarose gel. 5 µl HyperLadder 1KB (Bioline) was loaded into the first and last lanes of the gel to determine DNA molecular weight. Agarose gel electrophoresis conditions were 150 v for a maximum of one hour, before imaging on a Bio-Rad Gel Doc EZ Gel Imager (Image Lab 3.0).

**Bioanalyzer chip-based electrophoresis protocol**

Chip-based electrophoresis (Bioanalyzer) was applied to FFPE samples due to difficult detection from agarose gel electrophoresis, plus the inability to accurately detect fragmentation and molecular damage. Firstly, the Bioanalyzer (Agilent 2100) was cleaned with 350 µl dH_2_O using an electrode cleaner. The DNA chip was added to the chip priming station and 9 µl of gel dye mix (DNA dye concentrate and DNA gel matrix) was dispensed into wells. 1 µl sample or reference ladder was pipetted into respective wells. DNA marker (5 µl) was also added into all sample wells, plus one ladder only well as a reference. The loaded DNA chip was vortexed (IKA Vortex mixer) at 2400 rpm for one minute and loaded into the Bioanalyzer receptacle for 40 minutes. Sample concentrations (pg/µl) and molecular size (bp) were analysed by peak tables and electropherogram gel images (2100 Expert).

**Illumina amplicon sequencing protocol**

Illumina amplicon sequencing was performed by Northumbria University (Newcastle) as described within the main body of the manuscript, using the universal 16S rRNA gene primer specific to the V4 region (V4F: 5’-GTGCCAGCMGCCGCGGTAA-3’, V4R: 5’-GGACTACHVGGGTWTCTAAT-3’; Biesbroek *et al*, 2012). Firstly, 17 µl AccuPrime PFX SuperMix was added to each well of a 96-well plate, as were 2 µl of each paired set of primers (10 µm). 1 µl of sample, wax or kit negative control was transferred into corresponding wells. Each plate also contained one well of negative control (dH_2_O). Plates were briefly vortexed before DNA was amplified in a thermal cycler (Applied Biosystem, 2720) according to PCR cycling conditions of: initial denaturation at 95 °C for two minutes, followed by 30 cycles of denaturation at 95 °C for 20 seconds, annealing at 55 °C for 15 seconds and extension at 72 °C for five minutes, plus a final extension step at 72 °C for ten minutes. A 1% agarose gel was prepared to confirm successful amplification. Nextera XT index primers were added and amplified via PCR as previously.

The product was purified using AMPure XP bead by centrifuge the PCR plate 280 xg for one minute at 28 °C to collect condensation. 56 µl of AMPpure XP beads were added to each well of the PCR place, mixed and incubated for five minutes. The plate was then transferred onto a magnetic stand (Invitrogen) for two minutes, until the supernatant had cleared. The supernatant was discarded, and beads were washed using 200 µl of 80% ethanol and incubating for 30 seconds. The wash step was repeated twice whilst the plate remained on the magnetic stand. Following the final wash, the bead were allowed to air dry for ten minutes. Once removed from the magnetic plate stand 27.5 µl of 10MM TRIS PH 8.5 was added to each well of plate and incubated for two minutes, before transferring 25 µl to a new 96 well PCR plate. Library quality and optimum dilutions were assessed by chip-based gel electrophoresis following dilution of pooled libraries to 1:10, 1:100, 1:1000, 1:2000 and 1:4000 in PCR grade H_2_O. Results were used to calculate library concentration, with an optimum dilution selected. Libraries were quantified by qPCR with 6 µl KAPA SYBR Fast qPCR mastermix, plus 4 µl sample dilutions or standards in triplicate. Plates were vortexed before amplifying in a thermal cycler (C100; Bio-Rad) with the conditions of 95 °C for five minutes for initial activation, followed by 35 cycles of denaturation at 95 °C for 30 seconds and annealing at 60 °C for 45 seconds. 5 µl of eluted DNA from half of the wells were pooled and repeated for the other half of the plate. Positive sequencing controls were prepared, including a bacteriophage PhiX genome at 500 bp used as an in-run control for quality sequencing (2000ng, diluted 1 in 10, ZymoBIOMICS), and 10 µl 0.2 NaOH was added to pre-prepared libraries. All were incubated for five minutes to denature the DNA, before library preparations and 15% PhiX were mixed and loaded onto the flow cell (600 µl). Forward and reverse sequence primers (3 µl), plus index primers (3 µl) were added to three independent wells. The flow cell was washed with dH_2_O and blotted dry, before adding the reagent cartridge and flow cell to the MiSeq.

**Reference**

Biesbroek G, Sander EAM, Roeselers G, Wang X, Caspers MPM, Tezcinski K, Bogaert D, Keijser BJF. Deep sequencing analyses of low-density microbial communities: Working at the boundary of accurate microbiota detection. PLoS One. 2012; 7 (3) e32942 DOI: 10.1371/journal.pone.0032942.
